# The Need for a Rural Surgeon Speciality Pathway in Australia and New Zealand

**DOI:** 10.1111/ajr.70053

**Published:** 2025-05-06

**Authors:** Gavin J. Carmichael, Joshua G. Kovoor, Brandon Stretton, Alexander Beath, John Kefalianos, Daksh Tyagi, Connor Bentley, Stephen Bacchi, Jonathan Henry W. Jacobsen, Weng Onn Chan, Aashray Gupta, Ammar Zaka, Silas D. Nann, Yuchen Luo, Matthew Marshall‐Webb, Mathew O. Jacob

**Affiliations:** ^1^ University of Melbourne Melbourne Victoria Australia; ^2^ Grampians Health Ballarat Victoria Australia; ^3^ Grampians Research Initiative (GRIT) Ballarat Victoria Australia; ^4^ University of Adelaide Adelaide South Australia Australia; ^5^ Health and Information Australia; ^6^ University of Sydney Sydney New South Wales Australia; ^7^ University of Newcastle Newcastle New South Wales Australia; ^8^ St Vincents Hospital Fitzroy Victoria Australia; ^9^ Harvard Medical School Boston Massachusetts USA; ^10^ Royal Adelaide Hospital Adelaide South Australia Australia; ^11^ Gold Coast University Hospital Southport Queensland Australia; ^12^ Austin Health Heidelberg Victoria Australia; ^13^ Northern Health Epping Victoria Australia; ^14^ Flinders University Adelaide South Australia Australia

## Abstract

**Aims:**

This commentary aims to address the critical shortage of surgeons in rural Australia and propose the development of a sustainable rural surgical training pathway. By examining current healthcare disparities and workforce challenges, it highlights the need for locally trained and retained rural surgeons to improve health outcomes and reduce healthcare inequities.

**Context:**

Rural Australians experience significant healthcare disparities due to geographical isolation, lower socioeconomic status and limited availability of specialist care. The current model relies heavily on patient transfers to metropolitan centres, which are costly, logistically challenging and unsustainable. Current surgical training programmes offer some rural exposure; however, they remain metropolitan‐centric, resulting in fewer surgeons practising in rural areas.

**Approach:**

A dedicated rural surgical training pathway is proposed to address this gap. It would focus on selecting candidates with a demonstrated commitment to rural practice and provide tailored training, mentorship and guaranteed rural placements. Training must align with the specific healthcare needs of rural communities. Additionally, initiatives like the rural health equity strategy and regional training hubs must be supported by structural changes in the selection process to prioritise rural trainees.

**Conclusion:**

Addressing the shortage of rural surgeons is essential to improving healthcare equity. A rural surgical training pathway can aid in long‐term retention of surgeons in rural areas. This model supports both healthcare and economic sustainability, aligns with national rural health strategies and fosters stronger community connections. Investing in rural surgical training is a critical step towards reducing healthcare disparities and building a more resilient rural health system.

**Summary**

- Rural Australians experience critical disparities in surgical access, worsened by metropolitan‐centric training pathways.
- A dedicated rural surgical training program selecting for rural commitment and guaranteeing rural placements is essential for workforce sustainability.
- Investing in rural surgical training improves healthcare equity and drives economic and community development in rural regions.

Geography is destiny – Napoleon Bonaparte [1]

Australia's vast landscape presents unique challenges and opportunities for healthcare delivery in rural and metropolitan areas. Rural areas are characterised by their expansive geography, lower population density and significant distances between towns and tertiary medical facilities. This contrasts sharply with metropolitan areas, which are densely populated and have a high concentration of healthcare resources. The vast distances in rural regions can lead to challenges in accessing timely and comprehensive medical care, impacting overall health outcomes. Access to well‐trained surgeons is a significant part of this dilemma; to address this, a rural surgery training pathway is necessary.

## 1 Disparities Exist Despite Current Technologies

The demographic profile of rural areas differs significantly from that of metropolitan areas. According to the Australian Bureau of Statistics (ABS) [2], rural populations tend to have an older average age compared to their metropolitan counterparts and are less likely to have completed Year 12 or attained non‐school qualifications. This educational disparity can affect health literacy and access to employment opportunities, further influencing health outcomes. In terms of income, rural residents typically have lower average incomes but face higher costs for goods and services, exacerbating economic challenges and limiting access to healthcare [2].

Health behaviours and outcomes further highlight the divide between rural and metropolitan areas. Health behaviours such as smoking show a marked increase with greater remoteness [2]. Further, rural patients encounter greater barriers to successful surgical recovery due to geographical isolation, surgery away from home and less access to intensive rehabilitation [3]. Additionally, the total burden of disease and injury is higher, leading to higher rates of potentially avoidable deaths and lower life expectancy [2]. Residents of very remote areas are 1.9 times more likely to be hospitalised than those in major cities [2], reflecting the compounded health risks associated with rural living.

The current solution to providing safe surgical care to those in rural areas has been the collaboration between rural and metropolitan healthcare centres. These partnerships, based on catchment areas, involve the transfer of patients from rural to metropolitan centres for specialised treatments and surgeries. Improved connectivity has also meant the provision of telemedicine consults and clinics, allowing for consultations and follow‐ups without the need for travel. The expense related to transfers for routine surgical care, however, is a heavy strain on an already burdened system.

Collaboration provides a temporary solution, but it does not address the root cause of the healthcare disparity: the shortage of trained surgeons in rural areas [4]. A sustainable solution is necessary to improve healthcare outcomes, reduce dependency on metropolitan centres, and, most importantly, have advocacy for a rural cohort of patients. Our focus should be on training and retaining more surgeons within rural areas [4]. Increasing the number of rural‐based surgeons not only ensures that patients have access to timely and specialised care locally but is also an integral aspect of building infrastructure capable of meeting the unique challenges of rural health for the future.

Surgeons, once established in remote and regional areas, play a significant role in health advocacy within their community. A prime example is the work of Dr Ollapallil Jacob in Alice Springs. When he started working in Alice Springs, there was an astoundingly high burden of vascular trauma. Research conducted by his team demonstrated high levels of stab injuries per capita, roughly 390 per 100 000 people [5], resulting in Alice being known as ‘the stab capital of the world’ [6]. However, through dedication to his community, leadership, research and government lobbying, Dr Jacob drove policy changes that significantly reduced these numbers [7]. Ultimately, surgeons should not be underestimated in their ability to create greater change and importance within the communities they serve.

Building on this, it is essential to recognise the role of rural surgical training in cultivating the next generation of surgeons equipped to make impactful contributions. Recent research has demonstrated that students who undertake rural training have a higher five‐ and eight‐year retention rate in a rural centre compared to their metropolitan counterparts [8]. Additionally, general surgery residency graduates are more likely to choose rural hospitals or practices if they have a rural background [9]. Therefore, there is a strong case for developing a rural surgeon training programme and pathway.

## 2 Current Surgical Training Programs

The most recent selection criteria for general surgery training included Situational Judgement Test, Curriculum Vitae and Rurality, with points awarded for rural origin, rural medical school experience and rural pre‐surgical education & training work exposure. The General Surgery Education & Training program (GSET) currently faces a bottleneck in selection, with a skew towards metropolitan applicants [10]. Certain aspects of selection and training may divert students and junior doctors towards urban areas. Although 30% of medical students initially aim to practice in rural areas, only 5% ultimately specialise there, highlighting a significant gap despite efforts to incentivise rural exposure [11]. The Royal Australasian College of Surgeons' (RACS) are trying to address this with the ‘Rural Health Equity Strategy’ [10]. This initiative would consist of quarantined positions with applicants selected based on rurality criteria. Another initiative is the Northern Territory (NT) Rural Training Pathway [12], which aims to establish a longitudinal training pathway by selecting junior doctors who have or are already living, working and committed to remote areas. The programme focuses on providing extensive training opportunities within the NT to foster stronger connections to the region. The overall aim of this initiative is to address workforce shortages by retaining surgical trainees upon the completion of their training. This programme has already been implemented, with the first cohort of trainees set to commence in 2026.

In Victoria, through the Rural Health Multidisciplinary Training (RHMT) programme, the Western Victoria Regional Training Hub (WVRTH) was developed, which has a well‐established surgical training programme with rotations through Geelong, Ballarat, Hamilton and Warrnambool hospitals. Anecdotally, it has been stated that, as of 2023, this programme has a 100% fellowship exam pass rate, compared to 65.8% Australia‐wide [13]. However, with the current GSET selection process, rural applicants cannot specifically apply to this training programme. Instead, they apply centrally to RACS, with applicants being selected and then allocated through a preferencing system to the various training hubs, including the WVRTH. This leads to a skew towards metropolitan applicants getting these positions over rural GSET trainees. To address the imbalance and ensure that both rural and metropolitan areas have adequate access to trained surgeons, establishing separate metropolitan and rural general surgery streams, like the Rural Selection Initiative, could be beneficial. Such a training programme could empower rural communities by providing them with locally trained and retained surgeons.

## 3 Training and Retaining Rural Surgeons

A key aspect of any training programme is ensuring adequate speciality exposure. This becomes particularly important when considering a rural surgical stream, as rural surgeons require a broader base of skills compared to their metropolitan colleagues, given the limited access to subspecialists in rural institutions [14, 15]. In 2022, post‐fellowship training in rural surgery was introduced to address this need, aiming to equip surgeons with the diverse skillset required for rural practice. However, the programme was phased out in 2024 following a 2‐year period, with one successful applicant to complete the programme. At present, there are no post‐fellowship rural training pathways, with all advanced training opportunities remaining in metropolitan centres.

To address this, a sustainable rural post‐fellowship programme will require appropriate candidate selection, active support from speciality colleges and structured mentorship from a dedicated rural training committee. Candidates should receive formal recognition of training through an exit exam to uphold high standards of competency and excellence. Fellows who commit to such training deserve acknowledgement with a nationally recognised qualification, ongoing continued medical education and inclusion in metropolitan educational activities (e.g., journal clubs and local meetings). The program's oversight should be from members of the rural and regional surgical community who can invest time to ensure the training requirements of the fellow are being met. Surgical communities and speciality centres should be aware of the formal programmes prior to implementation, as this would allow for better support and engagement in training candidates.

## 4 A Sustainable Model

The authors believe a consideration should be placed on combining a post‐fellowship programme with a rural surgical stream, i.e., a surgical trainee who is guaranteed post‐fellowship training on entry into the rural surgical programme. This would allow for longitudinal guidance, candidate satisfaction, time to facilitate post‐fellowship specialisation and to plan for the set up of their practice towards the end of training. Longitudinal guidance is an important factor for regional doctors [16] and surgeons; the ability to build trust is based on exposure and time in the system. Candidates who have certainty in where they are placed, job security and know what their next years of training involves [17, 18] can then concentrate on acquiring the necessary surgical skills and niches of the subspeciality required in the community. It would also allow the trainee more certainty in planning life events and allow for greater investment in their future community. Guaranteeing subspeciality exposure as part of their post‐fellowship experience from entry into the surgical programme would allow the training committee time to identify and allocate appropriate locations for training years in advance.

## 5 An Economic Argument

Developing a rural surgical training pathway presents a compelling opportunity to enhance economic outcomes across multiple levels – patient, community, system and government. At the patient level, improved local access to surgical care reduces the need for travel and accommodation expenses, which are often significant burdens for rural and regional households [19]. By minimising indirect costs such as time away from work and disruption to family life, the pathway alleviates economic and emotional stress. Enhancing access supports overall financial stability for those residing in rural communities by reducing the overall costs associated with seeking care in distant locations.

At the community level, the indirect benefits of a rural training pathway extend beyond simply adding more trainees to the workforce. By training individuals with rural backgrounds or interests in rural areas, the program increases the likelihood that these doctors will remain in these communities [8], thereby reducing reliance on costly locum services, which only offer temporary solutions. The presence of stable healthcare services drives investment in local infrastructure and makes rural areas more attractive for long‐term settlement. The economic contributions of healthcare workers and their families, combined with population retention, create a positive feedback loop that supports local businesses, schools and community institutions, driving sustained economic growth.

At the health system level, the indirect economic benefits are realised through cost efficiencies and improved resource allocation. Decisions to transfer patients from rural and regional hospitals are inherently complex, requiring extensive knowledge of the local hospital's resources and capacity [20]. Employing locally trained surgeons provides the expertise necessary to navigate these complexities and potentially reduce costly and logistically challenging transfers to larger centres. Locally available surgeons can also provide timely interventions, preventing complications and the escalation of conditions caused by delayed treatment. Such delays and complications can increase hospital length of stay. For context, the basic cost of an overnight hospital bed is $751 [21], excluding additional investigations and procedures. Any complications or escalations due to delays in treatment impose a substantial financial burden on the healthcare system. Therefore, investing in rural training programmes is essential to ensure staff are retained in rural and regional areas, enhancing cost efficiency and sustainability within the local healthcare system.

The implementation of such a programme supports national and state‐level goals of investing in regional areas, addressing rural inequities and ensuring equitable healthcare access. By addressing systemic workforce shortages through locally rooted solutions, the programme aligns with national and state‐level goals while also attracting funding and resources for further infrastructure improvements. This alignment not only strengthens rural healthcare systems but also promotes broader economic development, making it a fiscally responsible and socially impactful initiative.

## 6 Conclusion

The tyranny of distance and geographic isolation, low population density and socioeconomic challenges leave rural regions underserved in surgical care. While telemedicine and collaboration provide interim support, addressing the shortage of rural surgeons is imperative. Dedicated programmes at all levels of training and selection criteria tailored to rural settings, such as the Rural Selection Initiative, are crucial steps in retaining and training the right type of candidates for rural surgery. Future research should focus on longitudinal retention studies and economic analyses to support the implementation of these programmes.

## Author Contributions

G.J.C.: Conceptualisation; Investigation; writing – original draft; writing – review and editing. J.G.K.: Conceptualisation; Investigation; writing – original draft; writing – review and editing. B.S.: Investigation; writing – original draft; writing – review and editing. A.B.: Investigation; writing – original draft; writing – review and editing. J.K.: Investigation; writing – original draft; writing – review and editing. D.T.: Investigation; writing – original draft; writing – review and editing. C.B.: Investigation; writing – original draft; writing – review and editing. S.B.: Conceptualisation; writing – original draft; writing – review and editing. J.H.W.J.: Investigation; writing – review and editing. W.O.C.: writing – original draft; writing – review and editing. A.G.: writing – original draft; writing – review and editing. A.Z.: writing – original draft; writing – review and editing. S.D.N.: writing – original draft; writing – review and editing. Y.L.: writing – original draft; writing – review and editing. M.M.W.: writing – original draft; writing – review and editing; supervision. M.O.J.: writing – original draft; writing – review and editing; supervision.

## Ethics Statement

The authors have nothing to report.

## Conflicts of Interest

The authors declare no conflicts of interest.

## Data Availability

Data sharing is not applicable to this article as no new data were created or analyzed in this study.
